# Case report: Preimplantation genetic testing for infantile GM1 gangliosidosis

**DOI:** 10.3389/fgene.2024.1344051

**Published:** 2024-02-09

**Authors:** Valeria A. Zagaynova, Yulia A. Nasykhova, Ziravard N. Tonyan, Maria M. Danilova, Natalya M. Dvoynova, Tatyana E. Lazareva, Tatyana E. Ivashchenko, Elena S. Shabanova, Inna O. Krikheli, Elena A. Lesik, Olesya N. Bespalova, Igor Yu. Kogan, Andrey S. Glotov

**Affiliations:** D. O. Ott Research Institute of Obstetrics, Gynecology and Reproductology, Saint-Petersburg, Russia

**Keywords:** rare diseases, GM1 gangliosidosis, assisted reproductive technology, preimplantation genetic testing, GLB1, PGT-M, haplotype analysis

## Abstract

Ganglioside-monosialic acid (GM1) gangliosidosis (ICD-10: E75.1; OMIM: 230500, 230600, 230650) is a rare autosomal recessive hereditary disease, lysosomal storage disorder caused by mutations in the *GLB1* gene that lead to the absence or insufficiency of β-galactosidase. In this study, we report a case of a Russian family with a history of GM1 gangliosidosis. The family had a child who, from the age of 6 months, experienced a gradual loss of developmental skills, marked by muscle flaccidity, psychomotor retardation, hepatosplenomegaly, and the onset of tonic seizures by the age of 8 months. Funduscopic examination revealed a «cherry red spot» in the macula, which is crucial for the diagnosis of lipid storage disorders. To find the pathogenic variants responsible for these clinical symptoms, the next-generation sequencing approach was used. The analysis revealed two variants in the heterozygous state: a frameshift variant c.699delG (rs1452318343, ClinVar ID 928700) in exon 6 and a missense variant c.809A>C (rs371546950, ClinVar ID 198727) in exon 8 of the *GLB1* gene. The spouses were advised to plan the pregnancy with assisted reproductive technology (ART), followed by preimplantation genetic testing for monogenic disorder (PGT-M) on the embryos. Trophectoderm biopsy was performed on 8 out of 10 resulting embryos at the blastocyst stage. To perform PGT-M, we developed a novel testing system, allowing for direct analysis of disease-causing mutations, as well as haplotype analysis based on the study of polymorphic markers—short tandem repeats (STR), located upstream and downstream of the *GLB1* gene. The results showed that four embryos were heterozygous carriers of pathogenic variants in the *GLB1* gene (#1, 2, 5, 8). Two embryos had a compound heterozygous genotype (#3, 4), while the embryos #7 and 9 did not carry disease-causing alleles of the *GLB1* gene. The embryo #7 without pathogenic variants was transferred after consideration of its morphology and growth rate. Prenatal diagnosis in the first trimester showed the absence of the variants analyzed in the *GLB1* gene in the fetus. The pregnancy resulted in the delivery of a female infant who did not inherit the disease-causing variants in the *GLB1* gene.

## 1 Introduction

GM1 gangliosidosis (ICD-10: E75.1; OMIM: 230500, 230600, 230650) refers to rare diseases, constituting an autosomal recessive hereditary disease, categorized as a lysosomal storage disorder. It occurs as a consequence of mutations in the *GLB1* gene, which lead to absence or insufficiency of β-galactosidase, encoded by GLB1 (Jarnes Utz et al., 2017). This enzyme catalyzes hydrolysis of terminal β-galactosyl residues present in GM1 gangliosides, glycoproteins, and glycosaminoglycans.

The prevalence of this condition is estimated at about one case per 100,000 to 200,000 live births, with regional variations based on ethnicity. For instance, heightened occurrences observed in Brazil (1 in 17,000 births) and Malta (1 in 3,700 births). Additionally, there is a notable increase in incidence of GM1 gangliosidosis carrier status among specific populations, such as Roma (1 in 50), Cyprus (1 in 12), and Japan (1 in 10) (Rha et al., 2021).

Based on the age of disease onset, GM1 gangliosidosis is categorized into three clinical phenotypes: infantile, late-infantile/juvenile, and adult/chronic. The most common and severe is infantile form, often resulting in fatal outcomes (Tonin et al., 2019). Clinical symptoms in infantile form of the GM1-gangliosidosis type 1 (GM1G1) appear in the first 12 months of the child’s life, but can be detected prenatally. Examination of the fetus with GM1 gangliosidosis may reveal nonimmune hydrops fetalis, intrauterine growth restriction, and placental vacuolization, congenital dermal melanocytosis (Mongolian spots) (Regier et al., 2013). About 50% of affected children have cherry-red maculae, identifiable during an ophthalmological examination, which can help make the diagnosis (Lang et al., 2020). As the child grows older, muscle hypotonia, hepatosplenomegaly, generalized skeletal dysplasia of varying severity, developmental delay, nystagmus, strabismus, corneal opacities, vision and hearing loss, gingival hypertrophy, cardiomyopathy, coarse facial features, and seizure syndrome may appear. The infantile phenotype is terminal and results in death before the age of 4 years (Utz et al., 2015). Diagnosis of *GLB1*-related disorder in a proband is established by identifying two pathogenic (or likely pathogenic) variants of the *GLB1* gene and/or by detecting a significant decrease in β-galactosidase activity in leukocytes or peripheral blood fibroblasts. Only one method is required to establish a diagnosis; the other can be used in combination to confirm the diagnosis or, in some cases, to determine genotype-phenotype correlation (Regier et al., 2013). The *GLB1* gene is situated on the short arm of chromosome 3 and encompasses 16 exons. More than 200 pathogenic variants in the *GLB1* gene have been identified to date, most of which are located within exons 2 and 6. This genetic heterogeneity contributes to the clinical diversity of the disease and complicates the diagnostic process (Brunetti-Pierri et al., 2008; Kwak et al., 2015).

There are currently no established and efficacious treatment modalities for GM1 gangliosidosis. Therapeutic strategies are experimental in nature, primarily aimed at slowing the progression of the clinical manifestations of the disease, improving the overall quality of life and prolonging its duration. This is achieved by mitigating the levels of GM1 gangliosides, augmenting β-galactosidase enzyme activity, or introducing exogenous enzyme supplementation (Rha et al., 2021). In cases where the pathogenic alleles of the *GLB1* gene in parents are known, it is recommended to perform preimplantation genetic testing for monogenic disorder (PGT-M) of embryos and invasive prenatal diagnostics (IPD) to determine the possible inheritance of both pathogenic variants of the parents (Hofer et al., 2010).

The incidence of GM1 gangliosidosis in Russia has not yet been well studied. Today in Russia there are no clinical guidelines for diagnosis, treatment strategy, and prevention of this disease. Therefore, diagnosis of GM1 gangliosidosis, management of the patients, and pregnancy planning for families with a history of GM1 gangliosidosis remains challenging. Considering that the Russian population represents more than 150 nationalities and many ethnic subgroups, further research is required on the frequency of genetic variants causing severe monogenic diseases, which may be specific to certain ethnic cohorts and may facilitate molecular diagnosis of the disease, including the preconception stage.

In the present study, we report a case of a couple with genetic family history that had one child with infantile form of GM1 gangliosidosis and faced the need to terminate the second pregnancy due to the affected fetus. The use of the assisted reproductive technology (ART) combined with PGT-M for 2 variants in the *GLB1* allowed this family to give birth to a healthy child. In this article we additionally set an objective to analyze the data on the *GLB1* variants’ allele frequency distribution based on the information from ClinVar, GnomAD, and RUSeq databases.

## 2 Patients and methods

### 2.1 Patients

In 2021, spouses A. sought medical genetic consultation due to the presence of monogenic disorder in a child from their first pregnancy. It is noteworthy that their first pregnancy, which culminated in an uncomplicated natural delivery in 2015, resulted in the birth of a female neonate with a weight of 2990 g, a length of 49 cm, and an Apgar score of 8-9 points at birth. During the first 5 months of life the child showed normal growth and development. However, from the age of 6 months a gradual loss of developmental skills was observed, marked by muscle flaccidity, increasing hepatosplenomegaly, coarse facial features, skeletal abnormalities, psychomotor retardation, and the onset of tonic seizures by the age of 8 months.

Following a comprehensive examination, ophthalmoscopy revealed a dark red macular spot, commonly known as the “cherry red spot” sign. This finding, combined with the clinical presentation, raised suspicion of genetically inherited storage disorders (Lang et al., 2020), including Stargardt’s hereditary macular dystrophy, Tay-Sachs disease (GM2 gangliosidosis), neuraminidase deficiency, galactosialidosis, and Niemann-Pick disease.

The child underwent an enzyme diagnosis for lysosomal storage diseases at the Laboratory of Inherited Metabolic Disorders within the Federal State Budgetary Scientific Institution “Medical Genetic Scientific Center.” The results revealed a pronounced decrease in beta-D-galactosidase activity in peripheral blood leukocytes (4.7 nM/mg/hour, with a reference range of 98.3–323.9 nM/mg/hour). However, the activity of palmitoyl-protein thioesterase 1 in leukocytes remained within the normal range (102.9 nM/mg/hour, with a reference range of 27.0–100.0 nM/mg/hour). There were no other changes in the activity of lysomal enzymes.

**FIGURE 1:**
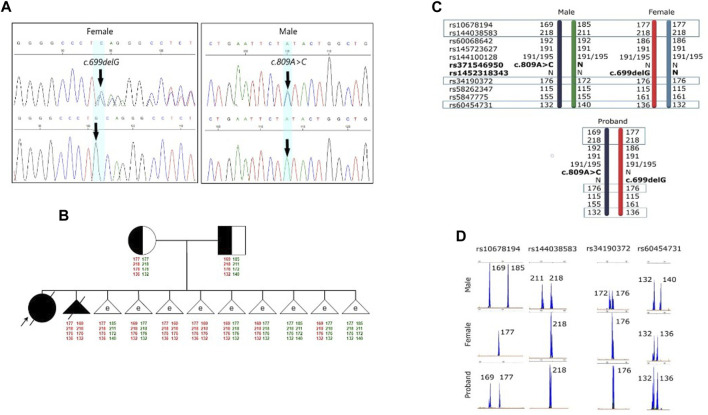
Results of the molecular genetic examination of the family with a history of GM1 gangliosidosis. **(A)** Results of molecular genetic examination of the couple showing heterozygous carriage of the variants in the *GLB1* gene. **(B)** Pedigree of the GM1 gangliosidosis family. Schematic diagram representing the STR-based haplotypes of family members and embryos. **(C)** Schematic diagram representing the STR-based haplotypes of parents and the proband. **(D)** Fragment analysis of four STR-markers of parents and the proband.

Further investigations were subsequently initiated to confirm the diagnosis of GM1 gangliosidosis, with an emphasis on mutation analysis of the *GLB1* gene. A single nucleotide deletion c.699delG (rs1452318343, ClinVar ID 928700) resulting in a frameshift and premature stop codon insertion was detected by direct sequencing in exon 6 of the *GLB1* gene in a heterozygous state. Additionally, a missense variant c.809A>C (rs371546950, ClinVar ID 198727) was found in exon 8 of the *GLB1* gene, also in a heterozygous state (Figure 1A).

Despite the administered treatment, the child’s condition continued to deteriorate, and she ultimately succumbed aged 2 years. The primary diagnosis was GM1 gangliosidosis with concomitant bilateral total pneumonia and organic brain damage.

### 2.2 Methods

#### 2.2.1 Assisted reproductive technology procedure and embryo trophectoderm biopsy

Ovarian stimulation was conducted in an IVF (*In Vitro* Fertilization) program utilizing recombinant FSH (rFSH, Gonal-F, Merck Serono, Italy) in a flexible protocol with gonadotropin-releasing hormone (GnRH) antagonists (antGnRH, Orgalutran, 0.25 mg, N.V. ORGANON, Netherlands). The total dose of rFSH administered was 1350 IU, and the stimulation lasted 9 days, with a total of 4 antGnRH injections.

When 3 or more follicles reached a diameter of ≥17 mm, GnRH agonists (Diphereline) were used at a dose of 0.2 mg to trigger ovulation. Thirty-six hours after the trigger for final oocyte maturation, transvaginal oocyte retrieval (TVOR) was performed, resulting in obtaining 18 oocytes-cumulus complexes. Fertilization through IVF method was assessed 17 h later, with 15 two-pronuclear zygotes, one single pronuclear zygote, and two three-pronuclear zygotes observed.

Embryos were cultured until the fifth day of development using Kitazato culture media (Japan) and assessed according to the criteria defined by Gardner and Schoolcraft (1999). Trophectoderm biopsy was performed on 8 out of 10 resulting embryos at the blastocyst stage, followed by their cryopreservation using Cryotop medium (Japan).

#### 2.2.2 gDNA extraction and WGA

The peripheral blood samples from the couple were collected in tubes with EDTA. Genomic DNA (gDNA) was extracted from peripheral blood leukocytes using the standard protocol for salt/chloroform DNA extraction with modifications (Müllenbach et al., 1989). Whole genome amplification (WGA) of the biopsied trophectoderm (TE) cells was performed using SurePlex WGA kit (Illumina) according to the manufacturer’s instructions.

#### 2.2.3 Pathogenic variant detection and STR analysis

To perform PGT-M, a direct approach was used to the study of pathogenic variants responsible for the monogenic disease in the family, and an indirect approach based on the analysis of polymorphic STR (short tandem repeats) markers located upstream and downstream of the *GLB1* gene. The primers were designed by means of Oligo 6 software and NCBI BLAST tool. The sequences of the primers used are given in Table 1.

**TABLE 1:** Primer sequences for analysis of disease-causing variants.

Gene	rs ID	SNP	Primer sequence (5′→3′)
*GLB1*	rs1452318343	c.699delG	F: GCC​TGT​GAT​TTT​GAC​TAC​CTG
			R: ACC​AAC​CTG​TTC​CAA​AGT​CCA
*GLB1*	rs371546950	c.809A>C	F: GCA​GTG​CAT​GTC​AGC​ATG​TCA
			R: CAA​GTT​CAC​ACT​CGC​CCC​ACG
*GLB1*	rs10678194	chr3:32920475–32920496	F: GAG​GAA​TGA​GTA​GAT​GGC​AGA
			R: GCA​AAA​GAA​AAG​AGG​CAA​GAA
*GLB1*	rs144038583	chr3:32934604–32934612	F: CTG​GCC​CTG​CAA​GGA​CAT​TAT
			R: TCC​CTT​CCT​GAG​TAT​CCA​TGA
*GLB1*	rs60068642	chr3:32963638–32963642	F: TGC​ACT​TAA​GAT​ATT​ATG​GAC
			R: GAA​CTT​TTT​TCA​AAT​TAC​CAA
*GLB1*	rs145723627	chr3:33026115–33026137	F: TCC​CCA​AGA​GTG​CAG​AGA​TGT
			R: GCA​GGA​AGG​AGG​TGG​TCA​GTC
*GLB1*	rs144100128	chr3:33049351–33049357	F: AGT​GAC​CAG​TTG​ATA​GGT​GTG
			R: ACC​AGG​TCT​CAA​CTG​TTT​CTC
*GLB1*	rs34190372	chr3:33058838–33058846	F: AAC​ATG​CTG​TGT​CTG​TCC​CTG
			R: TTA​CAC​AGA​CCC​ACA​CAC​TCC
*GLB1*	rs58262347	chr3:33123458–33123461	F: GCA​CTT​CAC​ATG​GCC​AGA​GAA
			R: ACC​ATC​CTC​TCT​GTG​CCA​TTC
*GLB1*	rs5847775	chr3:33143536–33143543	F: GCT​ATC​TCT​TTC​TCC​CTG​TAG
			R: CAG​AGC​TGT​CTA​GTT​TCA​TTC
*GLB1*	rs60454731	chr3:33202025–33202061	F: ATG​CTC​AGA​CTT​GTG​GGT​GCT
			R: CAC​CGT​TAA​CTC​CTT​GAG​AGA

The causal variants in *GLB1* were genotyped via polymerase chain reaction (PCR), followed by Sanger sequencing analysis. STR markers were analyzed using the microsatellite analysis method. Amplification conditions were as follows: initial denaturation at 95°C for 4 min; followed by 37 cycles of denaturation at 95°C for 30 s, primer annealing at 60°C for 30 s, elongation at 72°C for 30 s; and final extension at 72°C for 5 min. For variants determination, the products were purified by 5 M ammonium acetate, sequenced with ABI 3130xl (Thermo Fisher Scientific), and analyzed using SeqScanner Software 1.0 (Thermo Fisher Scientific).

## 3 Results

### 3.1 Medical genetic and reproductive physician counseling

To verify the carrier status of pathogenic variants previously identified in the child, genetic testing of the parents’ DNA by Sanger sequencing was carried out. It was discovered that the man is a heterozygous carrier of the likely pathogenic variant c.809A>C (rs371546950) in the *GLB1* gene, while his spouse was a heterozygous carrier of the pathogenic variant c.699delG (rs1452318343) in the *GLB1* gene (Figure 1A). There were no known cases of GM1 gangliosidosis or other hereditary diseases in their relatives. Standard cytogenetic testing revealed normal karyotype for both spouses (female: 46,XX, male: 46,XY).

The couple was advised to plan their pregnancy with ART and PGT-M on the embryos. Additionally, IPD in the first trimester of pregnancy was recommended to prevent the inheritance of the c.699delG and c.809A>C variants in the *GLB1* gene.

Subsequently, the couple sought consultation at the Assisted Reproduction Department of the D.O. Ott Research Institute of Obstetrics, Gynecology, and Reproductology with the aim of planning pregnancy through IVF for the application of PGT-M to embryos.

### 3.2 Pre-examination process

The pre-examination process included the development of testing system for detection of the 2 causal variants in the GLB1 gene and 9 indirect STR markers, the test validation using DNA samples and Whole Genome Amplification (WGA) products, and the informativity assessment of polymorphic STR-markers. Biological samples from all available family members were collected for analysis of the pathogenic variants’ segregation and haplotypes determination within the family. The biological material of the child from the first pregnancy (proband) was not available for analysis. During the indirect markers testing, the markers rs34190372, rs144038583, rs10678194, and rs60454731 were shown to be partially informative for this family and could be utilized for PGT-M. Due to the unavailability of the proband’s biomaterial, these markers, however, did not allow for performing the segregation analysis for STR-markers and the determination of the high/low-risk haplotypes within this family at this point, thereby increasing the testing process complexity.

During the pre-examination process period the couple had an unplanned natural pregnancy. At 11/12 weeks of gestation, chorionic villus sampling was performed to analyze fetal material for the presence of pathogenic variants in the *GLB1* gene. Prenatal diagnosis revealed that the fetus inherited both pathogenic variants in the studied gene. Due to these findings, the pregnancy was terminated at 12 weeks of gestation using a surgical method involving vacuum aspiration of the uterine contents. The postoperative period proceeded without complications.

Fetal material from IPD was used to complete haplotyping of polymorphic STR markers. The segregation analysis of polymorphic alleles identified one fully informative marker (rs60454731) and three partially informative markers (rs34190372, rs144038583, and rs10678194) for the family. The results of the family members’ evaluations are presented in Figures 1C, D.

### 3.3 Examination process

Biopsy samples of trophectoderm from 8 embryos were analyzed for the carriage of pathogenic variants c.699delG and c.809A>C in the *GLB1* gene. Additionally, the alleles of informative and partially informative STR markers, including rs34190372, rs144038583, rs10678194, and rs60454731, were assessed. The results of this analysis are presented in Table 2. According to our results, 4 embryos were heterozygous carriers of pathogenic variants in the *GLB1* gene, including 1 heterozygous carrier of c.699delG variant (#1) and 3 heterozygous carriers of c.809A>C (#2, 5, 8). 2 embryos had the compound heterozygote genotype (#3, 4). Embryos #7 and 9 inherited wild-type alleles of variants in *GLB1* (Table 2; Figure 1B).

**TABLE 2:** Detection of the pathogenic variants in the *GLB1* gene and haplotype analysis in embryos.

Еmbryo number/Blastocyst classification^a^	c.699delG	c.809A>C	rs34190372	rs144038583	rs10678194	rs60454731	Result
**1/4ВВ**	c.699delG/+	+/+	172/176	211/218	177/185	136/140	**Heterozygous carrier**
**2/5АА**	+/+	c.809A>C/+	176/176	218/218	169/177	132/132	**Heterozygous carrier**
3/5АА	c.699delG/+	c.809A>C/+	176/176	218/218	169/177	132/136	Compound heterozygote
4/5АА	c.699delG/+	c.809A>C/+	176/176	218/218	169/177	132/136	Compound heterozygote
**5/4АА**	+/+	c.809A>C/+	176/176	218/218	169/177	132/132	**Heterozygous carrier**
**7/5АА**	+/+	+/+	172/176	211/218	177/185	132/140	**Normal**
**8/4АА**	+/+	c.809A>C/+	176/176	218/218	169/177	132/132	**Heterozygous carrier**
**9/4АВ**	+/+	+/+	172/176	211/218	177/185	132/140	**Normal**

Bold font highlights the embryos that can be recommended for transfer.

^a^ According to Gardner D.K., Schoolcraft W.B., 1999 (Hofer et al., 2010).

### 3.4 Embryo transfer, prenatal genetic diagnosis, and pregnancy outcome

Four months later, the patient underwent the transfer of an embryo (#7) without disease-causing variants in the *GLB1* gene within a cryoprotocol employing estrogen and micronized progesterone as hormone replacement therapy. As a result of the treatment, the patient achieved a progressing intrauterine pregnancy with a single fetus.

At 11 weeks of gestation, chorionic villus sampling was carried out under ultrasound control. Cytogenetic analysis of the chorionic villus revealed a female karyotype (46,XX). Sanger sequencing confirmed the absence of the analyzed genetic variants.

The pregnancy progressed without complications and culminated in natural childbirth at 38-39 weeks of gestation, resulting in the delivery of a female infant weighing 3,100 g and height of 50 cm. The Apgar score for the newborn was 8/9 points. Both the mother and the newborn were discharged from the maternity hospital on the fifth day *postpartum*. At the time of reporting this clinical case, the child’s age was 3 months, her growth and development were consistent with age-appropriate milestones.

### 3.5 Assessment of the variant frequencies in the *GLB1* gene in Russian population

We made an attempt to estimate the allele frequency (AF) of pathogenic (P) and likely pathogenic (LP) *GLB1* variants causal to GM1 gangliosidosis in the Russian population. To do so, we have collected data on P and LP variants reported in NCBI ClinVar as at 28 October 2023 (Landrum et al., 2018). The association between variant and disease was discovered by the Orphanet identifier (ORPHA:354) as it describes all three clinical variants of GM1 gangliosidosis. AF of these variants were obtained from the only publicly available source of the AF in the Russian population, which integrates information about 7,492 exome samples, both from healthy donors (*n* = 1,945, according to Barbitoff et al., 2022) and diseased individuals with Mendelian/likely Mendelian/non-Mendelian phenotypes (with neurological and neuromuscular disorders prevalent in the most samples) (Barbitoff et al., 2021). In total, 12 out of 180 reported P/LP *GLB1* variants in ClinVar were identified in RUSeq data. Notably, 7 out of 12 P/LP variants were discovered only in exomes of diseased individuals. Global AF and AF in the non-Finnish European population for these P/LP variants were additionally received from the gnomAD (Table 3).

**TABLE 3:** Allelic frequency (AF) of pathogenic (P) and likely pathogenic (LP) *GLB1* variants associated with GM1 gangliosidosis in the Russian population.

rsID	Variation ID	RUSeq healthy population	RUSeq diseased donors	gnomAD global AF	Non-Finnish European population	Clinical significance^a^	Associated disease(s)^b^
rs398123351	92901	0.0002989	0	0.00002629	0.00004410	P/LP	GM1G2, GM1G3, MPS4B
rs794727165	194596	0.0002990	0	0.00001314	0.00001470	P	GM1G1, MPS4B
rs749980306	558213	0	0.0002086	0.000006573	0.00001470	P/LP	GM1G1, GM1G2, GM1G3, MPS4B
rs745386663	372371	0	0.0001044	0.00001971	0.00004409	P	GM1-gangliosidosis, MPS4B
rs376663785	284172	0.0002969	0.0004191	0.000006569	0.00001470	P/LP	GM1G1, GM1G2, GM1G3, MPS4B
rs1553610553	554850	0.0002964	0.0001043	0.00001314	0	LP	GM1G1, GM1G2, GM1G3, MPS4B
rs1452318343	928700	0	0.0001047	0.000006572	0.00001470	P/LP	GM1-gangliosidosis, MPS4B
rs72555366	939	0	0.0001044	0.0001315	0.00005882	P	GM1G 1, GM1G2, GM1G3, MPS4B
rs189115557	198077	0	0.0001049	0.00005919	0.0001029	P	GM1G1, GM1G2, GM1G3, MPS4B
rs778423653	554728	0	0.0001050	n.a	n.a	P/LP	GM1G1, GM1G2, GM1G3, MPS4B
rs778423653	417873	0	0.0001050	0.00001314	0.00001470	P/LP	GM1G1, GM1G2, GM1G3, MPS4B
rs72555392	945	0	0.0001043	0.000006571	0.00001470	P/LP	GM1G1, GM1G2, GM1G3, MPS4B
rs587776525	936	0.0002994	0.0001047	0.0001971	0.0001029	P	GM1G1, GM1G2, GM1G3, MPS4B

^a^ Clinical significance of genetic variant according to ClinVar as of 28 October 2023.

^b^ Associated diseases reported in ClinVar as of 28 October 2023; GM1G1, GM1-gangliosidosis type 1; GM1G2, GM1-gangliosidosis type 2; GM1G3, GM1-gangliosidosis type; 3MPS4B, Mucopolysaccharidosis type IVB.

## 4 Discussion

In this study, we report a case of the comprehensive examination of a couple with a family history of GM1 gangliosidosis, their pregnancy planning using ART with preimplantation testing of the pathogenic variants in the *GLB1* gene, and pregnancy management, which resulted in the birth of a healthy child.

Screening for carrier status of a monogenic disease in a couple, followed by PGT-M and/or IPD in case of increased risk detected is currently recognized as the most effective approach to reduce the risk of severe monogenic disease. However, some crucial aspects should be addressed in the management of such patients.

The availability of biological specimens from the proband, their parents, and other family members is critical for a testing system development and haplotyping. In our case, the sample from the proband was incorrectly stored in an external clinical organization. At the time the parents requested it for PGT-M, the sample was found to be not suitable for analysis, which resulted in a more complicated pre-examination process of PGT-M. Standardized collection and storage of clinical samples in biobanks can prevent the loss of important specimens due to improper storage. In addition, the validation of a new diagnostic system may require specimens with specific characteristics, which may also be resolved using biobank sample collections.

In our clinical case, patients had an unplanned pregnancy during the preparation for an IVF procedure with PGT-M. The presence of GM1 gangliosidosis in the fetus required medical termination of pregnancy. It is therefore essential to emphasize to patients the importance of contraceptive methods in the IVF program since it involves the transfer of the selected embryo. Additionally, in the case of pregnancy after IVF with PGT-M, confirmatory prenatal diagnosis in the first trimester is strongly recommended in order to avoid misdiagnosis caused by biological and human factors.

The low incidence and insufficient knowledge about the etiology and clinical manifestations of some rare hereditary diseases may limit the development of therapeutic strategies and early diagnostic approaches. Many monogenic diseases are characterized by ethnic differences in the prevalence and frequency of causative variants, which significantly complicate molecular diagnosis of these diseases especially among mixed populations, including the preconception period. The GM1 gangliosidosis risk was previously reported to be associated with ethnicity and genetic ancestry. The higher frequencies were shown for specific regions, such as Malta (Lenicker et al., 1997) and Brazil (Sperb et al., 2013). The founder mutation R59H (rs72555392) in the *GLB1* gene causing infantile GM1 gangliosidosis was previously described in an isolated population of Romani people in Bulgaria (Sinigerska et al., 2006). In Russia the genetics of GM1 gangliosidosis has not been sufficiently studied. In our study, we assessed the AF of P/LP variants in the GLB1 gene causal to GM1 gangliosidosis in the Russian population based on information from the new RUSeq database. In total, only 12 out of 180 reported P/LP *GLB1* variants in ClinVar were identified in RUSeq data. The variant rs1452318343 was reported in gnomAD, ClinVar, and RUSeq (cohort of diseased individuals) databases (Barbitoff et al., 2021). Previously it was found in multiple patients with infantile form of GM1 gangliosidosis from Ukraine (Mytsyk and Gorovenko, 2016). The variant rs371546950 was not described in any of the analyzed databases. Our results indicate significant ethnic differences in the GLB1 variant frequencies in Russia compared to other populations, which should be considered when developing diagnostic tools for genetic screening. Further research is required to determine the prevalence of the disease and the AF distribution in Russia.

## 5 Conclusion

Thus, PGT-M technology provides carriers of monogenic diseases an excellent opportunity to give birth to a healthy child and to avoid the risk for the pregnancy termination due to an affected fetus, which entails serious physical and psychological damage to patients. In this case we reported an ART combined with PGT-M for 2 variants in the *GLB1* gene, which allowed the couple with the genetic family history of GM1 gangliosidosis to give birth to a healthy child. However, such a complex and time-consuming medical procedure requires consideration of many aspects, some of which have been demonstrated in this case.

## Data Availability

The datasets for this article are not publicly available due to concerns regarding participant/patient anonymity. Requests to access the datasets should be directed to the corresponding author.
